# Quantitative evaluation of shoulder proprioception 6 months following stroke

**DOI:** 10.1186/s41983-018-0038-7

**Published:** 2018-11-06

**Authors:** Moshera Hassan Darwish, Sandra Ahmed, Ahmed Abdelalim, Abdelaziz Abdelaziz Elsherif

**Affiliations:** 1 Department of Neuromuscular Disorders and its Surgery, Faculty of Physical Therapy, Cairo University, Cairo, Egypt; 2 Department of Neurology, Faculty of Medicine, Cairo University, Cairo, 11562 Egypt

**Keywords:** Stroke, Shoulder, Proprioception

## Abstract

**Background:**

Chronic shoulder pain following cerebrovascular stroke (CVS) is a major problem that persists after maximum recovery of motor functions. Such pain has been attributed to altered shoulder joint kinematics causing soft tissue damage.

**Aim:**

Evaluation of shoulder proprioception in the ipsilateral paretic arm and contralateral unaffected side 6 months following cerebrovascular event.

**Subject and method:**

Thirty adult patients (G1) with ischemic strokes ranging from 6 months to 1 year and 30 healthy control (G2) were assessed for shoulder proprioception. Angular displacement error was measured during active and passive repositioning of shoulder external and internal rotation in both patients’ shoulders and in control’s dominant upper limb.

**Results:**

Statistically significant increase in angular displacement error was found in all tests in the affected shoulder compared to the unaffected contralateral shoulder and dominant arm of control subjects. The contralateral unaffected shoulder of patients showed within normal values and no differences with control values. Passive external and internal rotations showed statistically higher errors in patients with cortical lesions compared to those with subcortical lesions.

**Conclusion:**

Six months following the CVS, shoulder proprioception deficit in the affected hemiparetic side persists. Contralateral side shows no abnormalities. Cortical lesions might be associated with late shoulder proprioception recovery compared to subcortical lesions. The side of the lesion does not seem to affect the severity of proprioception deficit.

## Introduction

Stroke is a global problem with a burden that continues to increase due to population growth and aging [1]. Estimated prevalence of stroke in Egypt is 722/100,000 [2] with a higher prevalence among men and illiterate patients [3] which are by majority manual worker.

Improvement of quality of life (QoL) following stroke occurs up to 6 months following the event [4]. Shoulder pain is a major problem that occurs in nearly third of stroke patients with a majority ranging between moderate and severe degree. It appears around the fourth month [5] and persists in 21% of patients 16 months following stroke [6].

The sensorimotor system is defined as all of the sensory, motor, and central integration and processing components involved in maintaining joint stability [7]. Proprioceptive impairment as much as motor deficit is of outmost importance in planning of future management of stroke patients [8]. Chronic post-stroke shoulder pain (PSSP) has been attributed to altered kinematics of the joint leading to secondary soft tissue damage [9]. Rehabilitation of position sense showed promising results to avoid such problem [10].

The aim of this study is to evaluate shoulder proprioception in the ipsilateral paretic arm and contralateral normal side 6 months following the cerebrovascular event.

## Subjects and methods

This is a case-control study including 30 adults with anterior circulation ischemic cerebrovascular stroke patients (group 1) and 30 control healthy subjects (group 2) matching for age, sex, weight, and height. Patients were recruited from outpatient clinic of neuromuscular disorders and its surgery, Faculty of Physical Therapy, Cairo University. Included patients have stroke for more than 6 months and less than 1 year. The patients should have sufficient cognitive abilities to enable them understanding the requirements of the study. We excluded patients with frozen shoulder or any other orthopedic problems affecting the shoulders, language or hearing deficits, and diabetes mellitus. All patients had an almost equal physiotherapy program prior to the recruitment as they were following the standard program of the physical therapy department of neuromuscular disorders and its surgery, Faculty of Physical Therapy, Cairo University.

Both patients’ shoulders were assessed. The power of the affected arm ranged from 3 to 4+ according to “Medical Research Council Scale (MRC)” and spasticity from 1 to 1+ according to “Modified Aschworth Scale (MAS)” [11] to assure enough control of the patient over his range of movement. The control group (G2) was examined in the dominant shoulder only. According to brain imaging, patient’s lesions were subdivided according to site of the lesion into right or left and cortical (*n* = 5) or subcortical (*n* = 18). Patients with mixed cortical and subcortical lesions were excluded (*n* = 7).

### Primary outcome

The primary outcome is measuring the angular displacement error during active and passive repositioning of shoulder external and internal rotation. Absolute angular displacement error is defined as the difference in degrees between indicated and reference position during joint position sense assessment [12].

### Secondary outcome

The secondary outcome is finding a possible relation between persistent shoulder proprioceptive deficit and site of the brain lesion.

### Procedure

Individual shoulder proprioception assessment was done using “Biodex Isokinetic Dynamometer” and Biodex system III Multijoint testing and Rehabilitation System (Biodex, Medical Inc., Shirly, NY). Patient weight, height, and personal data were introduced in the system. Patient was seated with a back tilt 85°, dynamometer orientation 20°, and tilting 50°. Positioning of the patient was done so the axis of rotation of the fulcrum of the dynamometer is corresponding to axis of rotation of the shoulder. Two crossed straps were used to fix the patient to the chair in order to obtain isolated shoulder movement.

Absolute angular error was measured in the following two situations:

- *Passive reproduction of joint position (passive internal rotation and passive external rotation)*

Each patient was allowed to sense the reference angle of 10° for 10 s before performing the test. The tested arm was then passively moved in both directions subsequently, and the patient was asked to press a bottom with his untested hand if he feels that he reached the reference angle.

- *Active reproduction of joint position (active internal rotation and active external rotation)*

Same procedure as above was used but with the patient moving actively his limb to the reference angle relative to the starting position.

In each position reached by the patient, absolute angle displacement error was obtained by measuring the difference between the reference angle and indicated actual angle. Test was reproduced three times, and the mean of the three readings was considered final angular error.

Written consent was obtained from each patient after thorough explanation of the procedure, and the study was approved by the local ethical committee of Faculty of Physical Therapy, Cairo University.

## Statistical analysis

The statistical analysis was done using the Statistical Package of Social Science Software program, version 21 (SPSS, IBM Inc., Chicago, IL). The descriptive data were summarized using the arithmetic mean and the standard deviation for quantitative variable and the frequency percentage for qualitative variables. Comparison of the data within the same group was done using the paired *t* test and between groups using the unpaired *t* test.

## Results

Patient group (G1) and control group (G2) age, weight, and height were matched as seen in Table 1. There was male predominance in both groups with almost equal proportion (males/females) (G1; *n* = 23/7, G2; *n* = 21/9).

**Table 1:** Age, weight, and height of patients (G1) and controls (G2)

Variable*	Patients (G1)	Controls (G2)	*t* value	*P* value
Age (years)	55 ± 4.53	54.47 ± 4.36	0.4644	0.6441
Weight (kg)	84.4 ± 14.64	81.9 ± 15.37	0.6450	0.5215
Height(cm)	167.7 ± 7.9	165 ± 8.58	1.268	0.2098

*The values are expressed by mean ± SD

Comparison of angular displacement error in affected and unaffected shoulder of G1 showed a statistically significant difference (Table 2). Comparison of the affected shoulder of patients (G1) and the dominant shoulder of controls (G2) showed a statistical significant difference in all tests (Table 3). No difference was found between the unaffected shoulder of G1 and the tested shoulder of G2 (Table 4).

**Table 2:** Comparison of angular displacement errors of shoulder joint position sense in affected arm and unaffected arm of patients (G1)

Variables*	Affected arm	Unaffected arm	*t* value	*P* value
Active external rotation	3.823° ± 2.067	2.72° ± 2.141	2.03	0.0469**
Passive external rotation	4.597° ± 3.115	3.18° ± 2.384	2.178	0.0376**
Active internal rotation	4.573° ± 2.431	3.007° ± 1.802	3.339	0.0023**
Passive internal rotation	3.97° ± 3.546	2.657° ± 2.069	2.077	0.0468**

*The values are expressed by mean ± SD

**Statistically significant (*P* < 0.05)

**Table 3:** Comparison of angular displacement errors of shoulder joint position sense in the affected arm of patients (G1) and dominant arm of normal subject (G2)

Variables*	Patients (G1)	Controls (G2)	*t* value	*P* value
Active external rotation	3.823° ± 2.067	2.903° ± 1.378	2.028	0.0471**
Passive external rotation	4.597° ± 3.115	2.843° ± 1.959	2.610	0.0115**
Active internal rotation	4.573° ± 2.431	2.37° ± 1.402	4.301	0.0001**
Passive internal rotation	3.97° ± 3.546	2.327° ± 0.825	2.473	0.0164**

*The values are expressed by mean ± SD

**Statistically significant (*P* < 0.05)

**Table 4:** Comparison of angular displacement errors of shoulder joint position sense in the unaffected arm of patients (G1) and dominant arm of normal subject (G2)

Variables*	Patients (G1)	Controls (G2)	*t* value	*P* value
Active external rotation	2.72° ± 2.141	2.903° ± 1.378	0.3944	0.6948
Passive external rotation	3.18° ± 2.384	2.843° ± 1.959	0.5976	0.5524
Active internal rotation	3.007° ± 1.802	2.37° ± 1.402	1.527	0.1322
Passive internal rotation	2.657° ± 2.069	2.327° ± 0.825	0.8116	0.4204

*The values are expressed by mean ± SD

According to brain imaging, patients were subdivided into those with cortical lesions (*n* = 5) and patients with subcortical lesions (*n* = 18). Comparison of both subgroups showed a statistically significant increase in angular errors in patients with cortical lesions in passive external and internal rotation (Table 5).

**Table 5:** Comparison between patients with cortical and subcortical lesions in angular errors of the affected arm

Action*	Cortical (*n* = 5)	Subcortical (*n* = 18)	*P* value
Active external rotation	4.2° ± 2.38	3.56° ±2.38	0.88
Passive external rotation	5.8° ± 4.76	4.28° ±2.67	0.009**
Active internal rotation	4.4° ± 2.88	4.44° ± 2.43	0.408
Passive internal rotation	7.6° ± 6.54	3.2° ± 2.51	0.000**

*The values are expressed by mean ± SD

**Statistically significant (*P* < 0.05)

Subgroups with left, right, or bilateral lesions showed no statistical difference in degree of angular error of the affected arm in both active and passive external or internal rotations (*P* > 0.05).

## Discussion

This study demonstrates that shoulder proprioception deficit is an underestimated problem that persists beyond 6 months in paretic arm of stroke patients despite adequate physical therapy. This deficit may be more common in patients presenting with cortical lesions.

The upper limit of age for patients and controls was 63 years. Anatomical and physiological changes of the central and peripheral nervous system affect proprioception with advancing of age [13, 14]. We chose patients with stroke onset at least 6 months prior to inclusion period as maximum recovery of sensory functions usually occurs within this period [15] although continuous response to rehabilitation programs was shown to extend beyond this limit of time [16].

Patient group (G1) with cerebrovascular stroke (> 6 months) had an increased absolute angular displacement error in all tested positions (active external rotation, passive external rotation, active internal rotation, and passive internal rotation) when compared to unaffected contralateral arm and same aged control group (G2) (*P* > 0.05). Niessen et al. showed similar results when he compared proprioception of the shoulder in the paretic arm compared to controls [12]. Yet he showed that the contralateral arm as well was affected to some extent unlike our results that shows no affection of the contralateral side. This difference can be attributed to the time of testing; he chose patients with sub-acute stroke, but our patients were assessed 6 months following stroke. This time lapse can be sufficient for contralateral recovery but not for the paretic side. Normal function of the shoulder was stated to depend on shoulder stability; static stabilization that depends on capsulolabral complex and dynamic stabilization that depends on scapulo-thoracic and glenohumeral muscles both coordinated by neuromuscular control [17]. Gooey et al. [18] showed that the muscle spindle is the outmost important structure in determining joint position. The shoulder seems to contain very few mechanoreceptor [19] that is why Neissen et al. [12] said that the kinesthetic deficit in stroke patients is primarily due to gamma motor neuron dysfunction of muscle spindles sensitivity so there is a delay in detection of muscle stretch, thus creating the cascade of shoulder instability, soft tissue injury, and chronic pain [9].

Angular error of passive external and internal rotation of the shoulder was statistically higher in patients with cortical lesions (*n* = 5) compared to patients with subcortical lesions (*n* = 18) (*P* = 0.009 and 0.000 respectively). In a previous study, patients with cortical lesions were shown to recover more rapidly than patients with subcortical lesions, yet recovery was used in terms of motor function only [20]. Proprioception deficit seems to be different as it is more linked to cortical sensory integrating regions. Right supramarginal gyrus was proved to play a major role in proprioception loss in stroke patients [21] thus may be explaining prolonged recovery of proprioception in patients with cortical lesions. On the other hand, relation between lesion site (right or left) and proprioceptive deficit was rejected by Dukelow et al. [8], which support our results where no difference was found between site or bilaterality of the lesion and proprioception deficit. Yet the small number used in both studies could not give conclusive results.

## Conclusion

Chronic shoulder proprioceptive deficit persists beyond 6 months following cerebrovascular event but only in the affected paretic arm. This persistence may be linked to cortical lesions rather than subcortical ones. A new perspective in rehabilitation of stroke patients should include an extended program targeting deep sensory system as much as motor system through the first year following the event in order to achieve a better quality of life increasing independency and decreasing chronic debilitating pain.

## Abbreviation

CVS: Cerebrovascular stroke
MAS: Modified Aschworth score
PSSP: Post-stroke shoulder pain
QoL: Quality of life
